# Supplementary material for: Human Sinusoidal Subendothelial Cells Regulate Homing and Invasion of Circulating Metastatic Prostate Cancer Cells to Bone Marrow
Source: Cancers (Basel). 2019 May 31;11(6):763. doi: 10.3390/cancers11060763 (PMC6627911; doi:10.3390/cancers11060763)
Supplement: Supplementary file 1 [file cancers-11-00763-s001.pdf]

Supplementary Materials: Human Sinusoidal Subendothelial Cells Regulate Homing and Invasion of Circulating Metastatic Prostate Cancer Cells to Bone Marrow

Alessia Funari, Maurizio Alimandi, Luca Pierelli, Valentina Pino, Stefano Gentileschi, Benedetto Sacchetti

Supplementary Results

Prostate cancer metastases to bone marrow in humans.

To validate results of transplantation experiments, we compared them to observations made in humans nascent bone metastasis. Biopsies of prostate cancer metastases to iliac bone (Department of Pathology, University Sapienza of Rome) demonstrated focal seeding of cancer cells in marrow space. Using confocal fluorescence microscopy, cancer cells in situ, generated early cancer foci around vessels outer surface or in their close proximity, in association with hCD146^{+} mural cells.

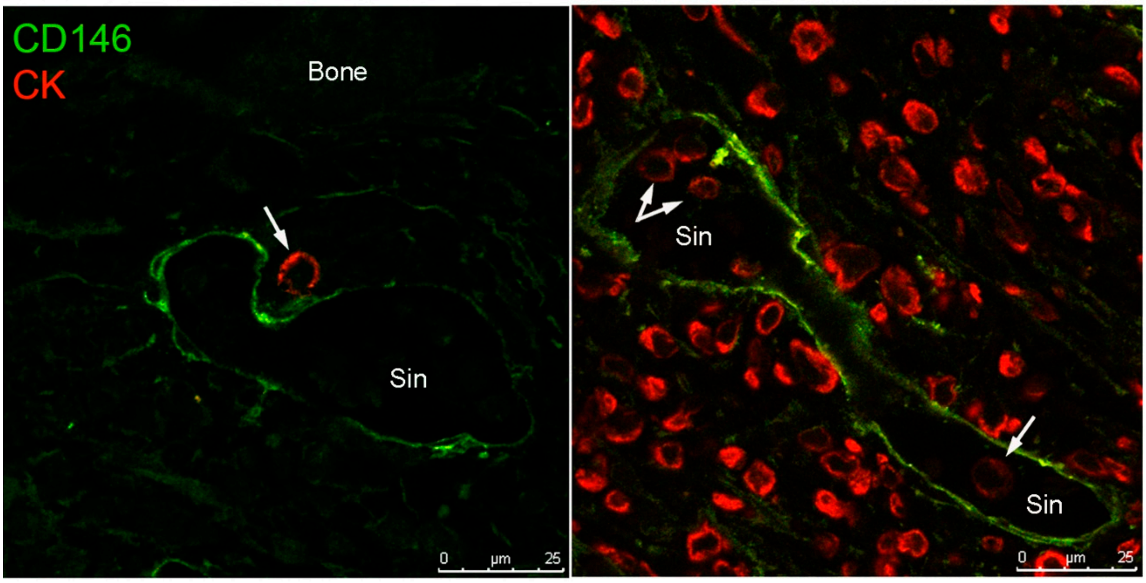

Figure S1. Early stages of prostate cancer metastases to humans bone marrow. Biopsy of prostate cancer metastases to iliac bone. High-resolution confocal image of human bone marrow biopsy specimen with the first metastatic foci of prostate adenocarcinoma cells. Perivascular mesenchymal stem cells expressing CD146 are green, while cytokeratin (CK, AE1/AE3, Abcam, Cambridge, UK) tumor cells, are in red. Scale bars represent 25 µm as indicated.
